# Diagnosis and Treatment of Traumatic Occlusion

**Published:** 1918-04

**Authors:** Clarence F. Brooks


### DIAGNOSIS AND TREATMENT OF TRAUMATIC OCCLUSION

BY CLARENCE F. BROOKS, D.D.S.

The familiar term "traumatic occlusion" took on a new significance for those present at a clinic given by Dr. Stillman, during his recent visit to Toronto. It has been, of course, common knowledge that a tooth in gross malposition, repeatedly wrenched and twisted by the movements of the mandible, was destined to unhappy experiences. But the fact that seemingly unimportant variations in the masticatory stress on the different teeth should seriously disturb the health of the periodontal tissues, was a decidedly new doctrine.

It is Dr. Stillman's belief that the correction of traumatic conditions is a prophylactic measure in the true sense of the word, because pyorrhoea will surely develop if the trauma is allowed to remain. Again, if a pyorrheal condition has developed, no treatment is complete which permits any of the teeth to remain subject to abnormal stress.

The diagnosis of a traumatic occlusion appears in some respects to be difficult and in others reasonably easy. In the general survey of the mouth it will, indeed, require the experienced eye to detect the horizontal line on the mucous membrane, some distance from the gingival margin. The margin itself may present a pressed-back appearance which is readily discernible. If the index finger is placed on the labial or buccal surface of the suspected tooth, and the patient instructed to exercise the normal movements of the mandible, as in mastication, the abnormal thrust is readily distinguished. A frequent example of this condition is that of a lower incisor exerting abnormal pressure in its occlusal relation with the upper incisor, the upper tooth being driven labially, and the lower lingually. Also, instructions may be given to perform the protrusive and lateral movements of the incisors edge to edge, the mandible to "catch," as it were, a malposed incisor or a long-cusped crown.

Dr. Stillman's treatment is characteristic and practical. Locate the exact area of over-stress and grind sufficiently to relieve the condition. By placing carbon articulating paper between the teeth, and resorting again to the "chopping" or other movements of the mandible as are indicated, the areas requiring attention are blackened. Another method is by the biting through of slightly warmed, thin wax instead of using articulating paper. The portions of the teeth showing through the wax are then blackened with a lead pencil, and the wax removed. To relieve the stress, grind with small stones on the exact area previously located. This may produce another malocclusion at some other point, which in turn must be corrected. The tooth, at a later date, may be found to have come again into malocclusion, and repeated grinding may be necessary in order to retain the tooth in normal coordination.

In cases where the wearing down of the teeth has been excessive, and large areas of dentine are exposed on the occlusal surfaces, it will be found that the wear of extensive metal or porcelain restorations has not kept pace with the wearing down of the dentine. These conditions are a fruitful source of trauma, and all such should be tested with carbon articulating paper, and corrected where indicated. This correction is necessary not only once, but at intervals sufficiently frequent to maintain proper occlusion.

A valuable hint was thrown out by Dr. Stillman regarding the relief of extreme sensitiveness of the cementum, during scaling operations. Take a bit of split bamboo which is hard on one side and has pith on the other, trim it thin and almost to a point, dip into 40 per cent. formaldehyde solution until saturated, and gently rub the hypersensitive area. Great caution must be observed that the bamboo be not dripping with the solution, or the gums will be injured by the formaldehyde.—*Oral Health.*

